# The Best Method of Disposal of the Dead

**Published:** 1884-09-20

**Authors:** Frederick Gundrum


### THE BEST METHOD OF DISPOSAL OF THE DEAD.

BY FREDERICK GUNDRUM, M.D.

**Abstract of paper read before Sanitary Convention at Ionia, Mich., Dec. 13, 1883.**

What method of disposal of the dead was first resorted to is not known, but within historic times methods have varied with different people, according to their religious sentiments. The ancients employed two principal methods, viz.: inhumation, or burying; and cremation, or burning. Rome and Greece burned, while China, Egypt and Judea buried in one form or another. As the belief in immortality spread throughout Judea, the stronger grew the attachment to inhumation, and the advent of Christ, together with his death and resurrection, gave the inhumation process the sanction of Heaven itself, and dealt a blow to cremation from which it has not recovered. The converts to the doctrines of Christ in Rome and Greece gradually abandoned burning, so that at the end of the fourth century there was but one method among civilized nations. With the feelings of individuals or nations, be they moral or religious, this paper does not deal: it runs in a different channel; viz., the sanitary aspect. The dead human body is subject to the same laws as other dead organic matter. Decomposition, more or less rapid, sets in according to the favoring circumstances of heat and moisture. In a few hours the deadliest poisons form, rendering quick disposition of the body absolutely necessary. The prevailing method of disposing of the dead will be first considered.

The first question is, how long shall a body be allowed to remain among the living? The cause of death should be considered an important factor. A person who has died of a highly contagious disease, such as diphtheria, malignant scarlatina, etc., should not be allowed to remain in the house longer than twelve hours. Such bodies ought to receive attention immediately after death by the plentiful use of disinfectants, etc., and placed in the coffin as soon as possible and not exposed again.

The rapidity of decomposition must be taken into consideration in those bodies not dying of contagious diseases. In hot, moist weather some bodies undergo very rapid change. The retention of such remains is extremely unwholesome, if not absolutely dangerous. Much has been done by undertakers, however, in arresting decomposition, and bodies can now be much longer retained than formerly. The use of solutions containing toxic agents should, however, be protested against. This method of arresting decomposition tends, and is likely, to cover up homicide, and thus shield the worst criminals known to the law. In Germany, France, Austria and other countries, preventive materials must be free from all poisonous agents. The lighter the garment, like the old-fashioned shroud, the more quickly will it succumb to the ravages of decay. The coffin should be made of the most perishable wood, and lead, zinc, etc., for coffins should be strongly condemned.

Various disinfecting and absorbing agents have been used with advantage, placed around the body to subdue the odor of decomposition, such as charcoal, tar, etc., mixed with sawdust or finely ground bark. Well dried, finely powdered peat is one of the best materials for this purpose.

The legislature has wisely interdicted the carrying of bodies of those dying of contagious diseases on trains or public funerals. It should, also, prohibit exhibiting the dead in churches and other public places.

The final resting place of the body should be sufficiently distant from the village or city not to interfere with the latter's growth.

An elevation should be selected. with forest trees, away from sources of drinking water, and out of the way of prevailing winds. Cemeteries are often started as private enterprises, regardless of fitness except as a financial basis. Committees are appointed by village boards and city councils. who locate cemeteries, not one of whom understands a single principle that should guide them in their responsible duty. This beautiful little city, if it continues to bury its dead in the new cemetery and use ice out of the old pond, will soon have the *pleasure* of drinking the percolations from their departed friends.

In times past graveyards were located in the heart of the town, around churches, and even under dwelling-houses; but the pernicious influence of this practice became recognized by the more enlightened people, who interdicted it by law. The Code Napoleon placed the distance at forty metres. Italy makes it two hundred metres. This is insufficient—the further the better. The earth should be dry and porous to be fit for burying purposes. In order to obtain a comparatively harmless and rapid decomposition, we must have a free circulation of air and sunshine.

It is evident that the danger from inhumation is by infecting air and water. Although the infection of the air is the lesser of the two evils, investigation has proven beyond a doubt that foul air has seriously influenced the health of individuals living near burial places. It is from contaminated water that the most serious results are found. Increased sickness and mortality in the vicinity of the old cemeteries of Paris and repeated chemical analyses prove this fact.

It is now known that disease germs may lie dormant in the ground for many years, waiting for a chance to reproduce disease and death. Knowing these facts, exhumation should not be permitted unless it is established that the individual died of a non-contagious disease. It should be made one of the duties of health officers to examine well and spring water, if used for drinking purposes, in the neighborhood of cemeteries, to see that it contains no organic matter.

The body burns, whether placed in the earth or fire. In one case it takes ten to twenty years, and in the other so many minutes. Cremation is the proper and scientific way to dispose of dead organic matter. When the body is cremated there is no further fear from disease germs in the body. The only plausible objection which has been offered against cremation is that, in case of homicide through the administration of deadly poisons, valuable evidence might be destroyed; but this is not a serious objection in the face of the many advantages gained. All innovations in sanitary science have had to fight their way inch by inch. Vaccination had a hard struggle, but came out triumphant, and so we predict for cremation a glorious victory, a triumph of good sense and science.—*Ionia Sentinel.*

---

THE valerianate of cerium is now suggested to replace the oxalate in vomiting of pregnancy.

